# Preparation and nanoencapsulation of l-asparaginase II in chitosan-tripolyphosphate nanoparticles and *in vitro* release study

**DOI:** 10.1186/1556-276X-9-340

**Published:** 2014-07-09

**Authors:** Elham Bahreini, Khosrow Aghaiypour, Roghayeh Abbasalipourkabir, Ali Rezaei Mokarram, Mohammad Taghi Goodarzi, Massoud Saidijam

**Affiliations:** 1 Research Center for Molecular Medicine, Hamadan University of Medical Sciences, Hamadan 6517619651, Iran; 2 Department of Genomics and Genetic Engineering, Razi Vaccine and Serum Research Institute (RVSRI), Karaj 3197619751, Iran

**Keywords:** Enzyme immobilization, Optimization, Ionotropic gelation, Nanoparticle, Cross-linking, Half-life

## Abstract

This paper describes the production, purification, and immobilization of l-asparaginase II (ASNase II) in chitosan nanoparticles (CSNPs). ASNase II is an effective antineoplastic agent, used in the acute lymphoblastic leukemia chemotherapy. Cloned ASNase II gene (*ansB*) in pAED4 plasmid was transformed into *Escherichia coli* BL21pLysS (DE3) competent cells and expressed under optimal conditions. The lyophilized enzyme was loaded into CSNPs by ionotropic gelation method. In order to get optimal entrapment efficiency, CSNP preparation, chitosan/tripolyphosphate (CS/TPP) ratio, and protein loading were investigated. ASNase II loading into CSNPs was confirmed by Fourier transform infrared (FTIR) spectroscopy, and morphological observation was carried out by transmission electron microscopy. Three absolute CS/TPP ratios were studied. Entrapment efficiency and loading capacity increased with increasing CS and TPP concentration. The best ratio was applied for obtaining optimal ASNase II-loaded CSNPs with the highest entrapment efficiency. Size, zeta potential, entrapment efficiency, and loading capacity of the optimal ASNase II-CSNPs were 340 ± 12 nm, 21.2 ± 3 mV, 76.2% and 47.6%, respectively. The immobilized enzyme showed an increased *in vitro* half-life in comparison with the free enzyme. The pH and thermostability of the immobilized enzyme was comparable with the free enzyme. This study leads to a better understanding of how to prepare CSNPs, how to achieve high encapsulation efficiency for a high molecular weight protein, and how to prolong the release of protein from CSNPs. A conceptual understanding of biological responses to ASNase II-loaded CSNPs is needed for the development of novel methods of drug delivery.

## Background

l-Asparaginase II (ASNase II) is an enzyme that is widely used for the treatment of hematopoietic diseases such as acute lymphoblastic leukemia. The enzyme is able to destroy asparagine-dependent tumors by degrading circulating l-asparagine and destroying malignant cells [1,2]. However, native ASNase II is associated with a high incidence of allergic reactions. Due to the formation of neutralizing antibodies, the half-life of circulating ASNase II (18 to 24 h) can be shortened to approximately 2.5 h [3]. Moreover, it is susceptible to proteolytic degradation by the proteases of the host organism. Much effort has been devoted to develop methods to avoid such side effects as well as to increase its *in vivo* half-life. For example, ASNase II has been chemically modified by polyethyleneglycol [4], poly-(d,l-alanine) [5], and dextran [6].

In the recent years, nanotechnology has shown a significant promise in the preparation of immobilized enzymes. Immobilization of enzymes onto biopolymer nanoparticles may result in some benefits, such as improving their stability to pH and temperature, as well as resistance to proteases and other denaturing compounds. Candidate carrier biopolymers should exhibit chemical and physical stability, biological compatibility, high purity, homogeneous molecular weight (MW) distribution, and adequate functional groups for binding to biomolecules with high loading capacity. They exhibit several drug loading mechanisms including electrostatic attractions, hydrophobic interactions, and covalent binding. They can form a matrix or membrane that can slow drug release over a prolonged period, avoiding repetitive dosing. However, one should bear in mind that covalent coupling of enzymes to polymers may result in conformational alterations, pharmacokinetic modifications, and a significant decrease in enzymatic activity. Examples of such biopolymer nanoparticles that ASNase II has already been incorporated in are liposomes [7], poly(d,l-lactide-co-glycolide) (PLGA) [8], and hydrogel-magnetic nanoparticles [9].

Chitosan (CS), produced by alkaline *N*-deacetylation of chitin, is another natural polymer that has good physicochemical (reactive OH and NH_2_ groups), as well as biological properties. It is composed of glucosamine and *N*-acetylglucosamine monomers linked by β [1-4] glycosidic bonds. CS is hydrophilic and soluble in acidic solutions by protonation of the amine groups. It is degraded by enzymes such as lysozymes, some lipases, and proteases. CS is a biologically safe, non-toxic, biocompatible, and biodegradable polysaccharide [10]. Current research with CS focuses on its use as a novel drug, gene, peptide, and vaccine delivery vehicle and as a scaffold for targeted drug delivery and tissue engineering applications [11,12].

Two groups of cross-linkers are usually employed to obtain CS particles. One group, such as glutaraldehyde and glucomannan, cross-links through covalent bonds leading to quite stable matrixes. The other group is ionic cross-linkers that cross-link through ionic gelation and electrostatic interactions between the positively charged chitosan chains and polyanions. The polyanion most commonly used for the ionic cross-linking is tripolyphosphate (TPP), which is non-toxic. Due to the proved toxicity of glutaraldehyde and other organic molecules used in the synthesis of gels covalently stabilized, only the second synthesis technique (ionic gelation) can be used for pharmaceutical applications.

Bodmeier et al. [13] and Calvo et al. [14] used an ionotropic gelation method to prepare CS particles with sizes ranging from micron to submicron for the first time, and this is a currently widely used method for preparing CSNPs. In this method, an anionic cross-linking agent is introduced into an aqueous solution of CS in acetic acid. The cross-linking structure of the CS/TPP system is mainly determined by the reaction between the amino groups of CS and TPP ions, and this reaction depends strongly on the associated pH [15,16]. Alteration in the parameters such as cross-linker concentration, drug/polymer ratio, and processing conditions affects the morphology of CSNPs and the release rate of the loaded drug [17,18].

Formulation development and optimization is a very critical process in the design and manufacture of any therapeutic drug. Depending on the design and delivery aims for a particular drug, the process requires several *in vitro* and *in vivo* study stages. Generally, the *in vitro* property is the rate or extent of drug dissolution and its release, whereas the *in vivo* study involves the plasma drug concentration, absorption, body interactions, and any possible side effects. The purpose of the *in vitro* study in the early stage of nanodrug development is to investigate the optimum formulation, evaluate the active ingredient, and assess any minor changes for drug development. The aim of the present work was to assess the *in vitro* preparation of ASNase II-loaded CSNPs cross-linked with TPP and to evaluate their efficacy for the entrapment and controlled release of the protein. The values were expressed as the averages of at least three independent experiments each.

## Methods

### Materials

The following materials were used: BL21 pLysS (DE3) strain (Novagen, Cat. No.: 69451–3, Darmstadt, Germany), pAED4 (BV Tech, Sofia, Bulgaria), isopropyl β-d-1-thiogalactopyranoside or IPTG (Sigma-Aldrich Cat. No.: I6758, St. Louis, MO, USA), Luria Bertani broth or LB broth (Merck, Cat. No.: 1.10285.0500, Whitehouse Station, NJ, USA), diethylaminoethyl (DEAE)-Sepharose Fast Flow (Amersham, Cat. No.: 17-0709-01, Amersham, UK), Sephadex G-75 (Sigma-Aldrich, Cat. No.: G7550), l-asparagine (Sigma-Aldrich, Cat. No.: A0884), Nessler's reagent (Sigma-Aldrich, Cat. No.: 72190), and CS (low molecular weight (% deacetylation 75% to 85%, viscosity 20 to 300 cP, average MW ~ 50 kDa), Sigma-Aldrich; Cat. No.: 448869), sodium tripolyphosphate (Sigma-Aldrich, Cat. No.: 238503).

### ASNase II production, extraction, and purification

According to our optimized protocol for overproduction of recombinant protein [19], ASNase II (EC 3.5.1.1) was expressed in transformed *Escherichia coli* BL21 pLysS (DE3). The periplasmic ASNase II was extracted from the bacterial pellet using modified alkaline lysis method [19]. The extract was clarified by centrifugation for 30 min at 30,000 × *g* at 4°C, and the supernatant was filtered through a 0.45-μm sterile filter. A single-step purification of ASNase II was performed by loading the filtrate sample onto the DEAE-Sepharose Fast Flow column (5 cm × 15 cm) pre-equilibrated with phosphate buffer (0.01 mM, pH 7.0). After removing the unbound proteins from the column by passing phosphate buffer, NaCl gradient from 50 to 200 mM was applied to the column at a flow rate of 4 ml/min. The collected fractions were analyzed for enzyme activity (U/ml) and protein content (mg/ml). The purity of ASNase II was judged using sodium dodecyl sulfate-polyacrylamide gel electrophoresis (SDS-PAGE) (15%) stained with Coomassie brilliant blue. The fractions with the higher ASNase II activity were pooled and analyzed for total activity (U), total protein level (mg), and specific activity (U/mg).

The purified solution from the previous step was desalted using Sephadex G-75 column (3.0 × 70 cm) pre-equilibrated with double-distilled water (DDW) at a flow rate of 3 ml/min. The most active fractions were pooled and concentrated by lyophilization (−50°C) and the protein powder was stored at 4°C.

### Estimation of ASNase II activity

The activity of ASNase II was assayed using the Nessler method [20]. A reaction mixture contained 0.5 ml Tris–HCl buffer (0.1 M, pH 8.5), 0.25 ml l-asparagine (10 mM in Tris–HCl buffer), and 25 μl of the enzymatic solution. After 15 min of incubation at 37°C, the reaction was terminated by the addition of 0.25 ml of 15% trichloroacetic acid (TCA). The liberated ammonia was determined by adding 0.25 ml of Nessler's reagent. The absorbance was recorded at 425 nm after 10 min. The absorbance values were converted to micromoles of ammonia using a standard curve prepared with ammonium sulfate. One unit of enzyme activity (IU) was defined as the amount of enzyme required to release 1 μmol of ammonia per minute under standard assay conditions.

### Estimation of protein concentration

Protein concentration was estimated with Folin phenol reagent (Lowry method) using bovine serum albumin as a standard [21].

### Preparation of CSNPs

CSNPs were prepared based on the ionotropic gelation method [22] with a small modification. The method is based on electrostatic interactions between the amine group of CS and the negatively charged group of TPP as a polyanion. During the process involving chemical reaction, CS undergoes ionotropic gelation and precipitates to form spherical particles that are distinguishable by opalescence of solution.

Low molecular weight CS was dissolved in DDW containing 1.2% acetic acid to a concentration of 0.5% (*w*/*v*) as stock solution. The isoelectric point of ASNase II and p*K*_α_ of CS are 4.9 [23] and 6.5 [24,25], respectively. The pH of the CS solution was adjusted to 5.7 by NaOH as the mean pH point. TPP with the concentration of 0.5% (*w*/*v*) in DDW was prepared as the stock solution. Both solutions were filtered through a 0.25-μm sterile filter.

### Preparation of ASNase II-CSNPs

#### ASNase II activity against CS and TPP

In order to determine the individual effect of each CS and TPP on ASNase II activity, 1 ml CS solution (0.2% (*w*/*v*), pH ~ 5.7) and 1 ml TPP solution (0.1% (*w*/*v*), pH ~ 8.5) were prepared from stocks. One milligram of lyophilized ASNase II was added to each solution, and both of them were slowly shaken for 15 min. The percentage of the preserved activity for both solutions was calculated based on the activity of untreated ASNase II (1 mg/ml), which was taken as 100%.

#### Two ways of preparation of the ASNase II-loaded CSNPs

The preparation of the ASNase II-loaded CSNPs via the ionotropic gelation method was examined in two ways. In the first approach, 1 mg of lyophilized protein was mixed with 1 ml of TPP solution (0.1% (*w*/*v*)), and the mixture was added dropwise to 1 ml of CS solution (0.2% (*w*/*v*)) with stirring using a magnetic stirrer. In the second method, 1 mg of lyophilized protein was mixed with 1 ml of CS solution (0.2% (*w*/*v*)), and TPP (0.1% (*w*/*v*)) was added dropwise to the protein/CS mixture with stirring. After 10 min of stirring, each opalescent solution was centrifuged (25,000 × *g*, 25°C for 30 min), and the amounts of the free protein in each supernatant were measured in order to analyze the entrapment efficiency.

### Optimization of CS and TPP concentrations

To optimize the CS/TPP ratio based on particle size and the entrapment efficiency, various CS concentrations (0.2%, 0.3%, and 0.4% (*w*/*v*)) were prepared from the stock solution. The concentrated TPP solution (0.5% (*w*/*v*)) was used in order not to dilute the CS/ASNase II mixture more than necessary. From this stock solution, different volumes of TPP solution (Table 1) were added dropwise (10 μl per 10 s interval) to 1 ml of each CS concentration (containing 1 mg lyophilized ASNase II) with stirring (about 800 rpm), with particular care taken to avoid foam formation. In addition to the applied volumes of TPP, Table 1 shows the final concentrations of the added TPP (% *w*/*v*). All procedures were carried out at room temperature (25°C). After 10 min of stirring, the particles were collected by centrifugation at 25,000 × *g*, 25°C for 30 min in 50-μl glycerol bed. The supernatants were separated to estimate the entrapment efficiency (%). The pellets of the particles in glycerol were suspended in 1 ml of distilled water to determine the average sizes (nm).

**Table 1:** **Chitosan concentrations, TPP volumes from TPP stock solution (0.5%** ***w*/ *v *), and final TPP concentrations in final prepared nanoparticle suspensions**

**CS (%** ***w*/ *v *)**	**TPP (ml)**	**TPP (%** ***w*/ *v *)**
0.2	0.1	0.04
	0.12	0.05
	0.14	0.06
0.3	0.15	0.06
	0.18	0.07
	0.21	0.08
0.4	0.2	0.08
	0.24	0.095
	0.28	0.11

### Optimization of protein loading

The stable and suitable CS/TPP ratio from the previous step was selected in order to investigate the optimal entrapment efficiency and loading capacity of CSNPs, loaded with five different amounts of protein (1, 2, 3, 4, and 5 mg). Nanoparticles were prepared according to the procedure given above by adding a certain amount of lyophilized ASNase II in 1 ml of optimal CS solution. After centrifugation, the supernatants were separated to estimate the entrapment efficiency. The pellets of the particles in glycerol were suspended in 1 ml of DDW and dispersed by sonication. The size (nm), zeta potential (mV), protein content (mg), entrapment efficiency (%), and loading capacity (%) of the particles were determined.

### Entrapment efficiency estimation

In order to determine the entrapment efficiency of the nanoparticles, it was necessary to detect by the Lowry method [21] the amount of free enzyme in the clear supernatant. The ASNase II entrapment efficiency was calculated using the following equation:

$$\begin{array}{l} \mathrm{Entrapment}\;\mathrm{efficiency}\left(\%\right)\;=[(\mathrm{Total}\;\mathrm{protein}\;\mathrm{used}\;\mathrm{in}\;\mathrm{formulation}\\ \;-\mathrm{Free}\;\mathrm{amount}\;\mathrm{of}\;\mathrm{protein})\\ \;/\mathrm{Total}\;\mathrm{protein}\;\mathrm{used}\;\mathrm{in}\;\mathrm{formulation}]\\ \;\times \;100 \end{array}$$

### Loading capacity estimation

The loading capacity was defined as the ratio of the amount of ASNase II entrapped and the weight of nanoparticles and calculated according to the following mathematical expression:

$$\begin{array}{l} \mathrm{Loading}\;\mathrm{capacity}\left(\%\right)=(\mathrm{Mass}\;\mathrm{of}\;\mathrm{protein}\;\mathrm{content}\;\mathrm{of}\;\mathrm{the}\\ \;\mathrm{nanoparticles}/\mathrm{Weight}\;\mathrm{of}\;\mathrm{nanoparticles})\\ \;\times 100 \end{array}$$

### Characterization of ASNase II-loaded CSNPs

#### Particle size and zeta potential of ASNase II-CSNPs

The particle size, size distribution (polydispersity index (PDI)), and zeta potential of particles were measured by Zetasizer (Malvern Instruments, Worcestershire, UK), based on the dynamic light scattering (DLS) technique. The mean particle size was approximated as the *z*-average diameter and the width of the distribution as the PDI. DLS measurements were performed at 25°C with a detection angle of 90°. All measurements were preformed in triplicate, and the results were reported as mean ± standard deviation.

#### Fourier transform infrared spectroscopy

Fourier transform infrared (FTIR) spectroscopy (Bruker, Ettlingen, Germany) was used to characterize bonding characteristics of the lyophilized ASNase II, CS, CSNPs, and ASNase II-CSNPs.

#### Morphological observations

Examinations of surface morphology and size distribution for CSNPs and ASNase II-loaded CSNPs were performed using a transmission electron microscope (TEM) (Philips CM30, Eindhoven, The Netherlands). About 5 μl of the nanoparticle solution was placed on a copper grid and stained with 2% (*w*/*v*) phosphotungstic acid.

### *In vitro* ASNase II release

ASNase II release from the matrix complex was evaluated in three solutions of glycerol (5%)-phosphate-buffered saline (PBS) solution (pH 7.4), PBS solution (pH 7.4), and DDW containing 5% glycerol (pH 7.0). ASNase II-loaded CSNPs with the highest protein loading capacity were suspended in each of these solutions and incubated at 37°C. At predetermining time points, nanoparticles were collected with a centrifuge (25,000 × *g*, 30 min and 25°C). The supernatant was removed for protein content assay.

The percentage of leakage from the nanoparticles was calculated using the following equation:

$$\%L=\;\left(M_{o}/\;M_{e}\right)\;\times \;100$$

where %*L* represents the percentage of leakage, *M*_*o*_ is the mass of ASNase II in the supernatant, and *M*_*e*_ is the mass of entrapped ASNase II.

### Effect of pH on enzyme activity and stability

The activities of the immobilized and free ASNase II were evaluated at different pH values in the range between pH 6.5 and 10 adjusted with Tris–HCl (0.1 M). In the case of pH stability experiment, the immobilized and free enzymes were incubated for 24 h at 4°C ± 1°C at different pH values (pH 6 to 10) in the absence of the substrate, and the residual activity was determined. The percentage of residual activities was calculated based on the untreated control activity, which was taken as 100%.

### Effect of temperature on enzyme stability

Thermostability studies were carried out by pre-incubating the immobilized and free ASNase II at different temperatures (37°C, 45°C, 50°C, 60°C, 70°C, 80°C, and 90°C) for 60 min, followed by cooling. The percentage of residual activities was determined and calculated based on the untreated control activity, which was taken as 100%.

### Half-life determination of the free and immobilized ASNase II

The solutions of Tris–HCl (0.1 M, pH = 8.5), DDW-glycerol (5%), and PBS-glycerol (5%) were considered for measuring the half-life of the free and immobilized enzyme. Solutions of the immobilized and free enzyme were slowly homogenized and incubated at 37°C to measure the half-life of both. At the time interval of 1, 3, 6, and 24 h, a sampling was done without replacement for the determination of enzymatic activity.

## Results and discussion

### Production and purification of ASNase II

**Figure 1:**
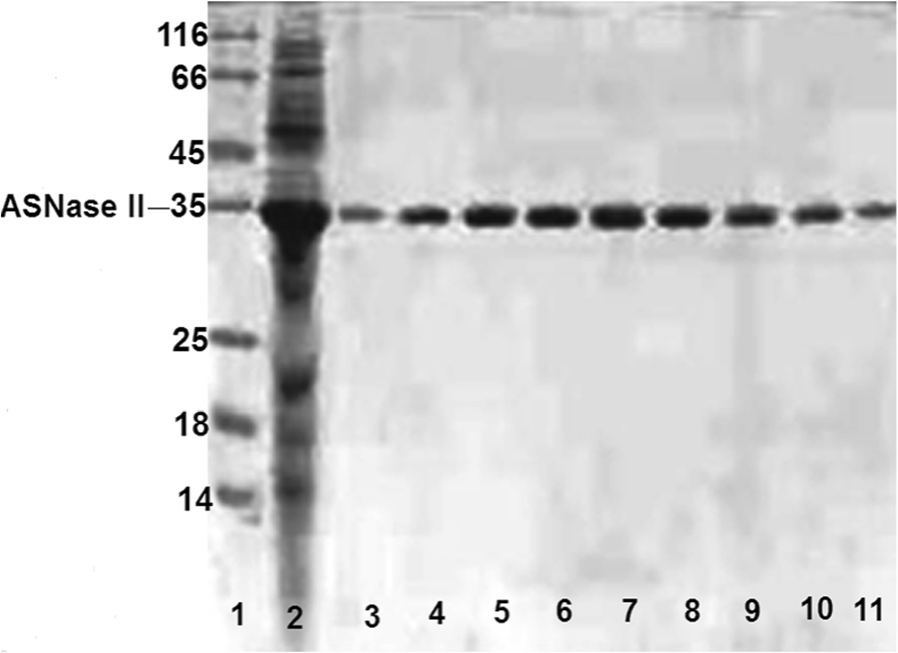
**SDS-PAGE (15%) analysis of ASNase II purification using DEAE-Sepharose.** Lane 1: protein marker. Lane 2: Crude extract of *E. coli* by alkaline lysis. Lanes 3 to 11: purified ASNase II eluted from the DEAE-Sepharose column in selected fractions.

As mentioned above, protein expression was carried out under conditions that were previously optimized in our laboratory. The extract prepared by alkaline lysis was passed through a DEAE-Sepharose Fast Flow column. Table 2 shows a summary of the results, before and after purification. The total specific activity increased from 18.6 to 111.5 U/mg for the filtrate and the final preparation, respectively. About 81.5% of the original enzyme activity was recovered with a purification fold of 6. Purification was examined by SDS-PAGE following Coomassie brilliant blue staining (Figure 1). It revealed only a single distinctive protein band for the pure preparation of ASNase II with an apparent molecular weight of 35 kDa, corresponding to a monomer of the denatured enzyme. All known types of ASNase II are active as homotetramers with molecular mass of approximately 140 kDa, arranged as 222-symmetric assemblies around three mutually perpendicular dyads. The closest interactions between the A and C subunits (as well as between subunits B and D) lead to the formation of two intimate dimmers within which the four non-allosteric catalytic centers are created. Such formation of tetramers, for reasons that are not completely clear, appears to be essential for the catalytic ability of ASNase II [26,27].

**Table 2:** Purification table of ASNase II by DEAE-Sepharose

**Steps**	**Volume (ml)**	**Total protein (mg)**	**Total activity (U)**	**Specific activity (U/mg)**	**Overall yield**^**a **^**(%)**	**Purification fold**
Before purification (filtrate)	80	786.4	14,604.48	18.57	100	1
After purification (DEAE-Sepharose)	187	106.7	11,896.8	111.5	81.4	6.0

^a^Yield = Total activity after purification/Total activity before purification.

Chloride (which would interfere with TPP in preparation of ionotropic nanoparticles) was eliminated from the DEAE-chromatographic product by Sephadex G-75 and the protein was lyophilized. At the high ionic strengths, the CS-TPP binding would be weakened to the point that the nanoparticles would cease to form [28], due to the competitive reaction between Cl^−^ and TPP ions for NH_3_^+^.

### Preparation of ASNase II-loaded CSNPs

#### ASNase II activity in CS and TPP solutions

Both CS and TPP have their characteristic charge and may likely affect ASNase II stability and activity. The behavior of ASNase II in the CS and TPP solutions was individually investigated before preparation of nanoparticles. The percentages of the preserved ASNase II activity in CS and TPP were 85% and 80% of the activity of untreated enzyme, respectively. This result can be explained from the standpoint of pH. As will be mentioned below, the optimum pH for the activity of free ASNase II is between 8.5 and 9 and enzyme activity decreases to about 86% at pH ~ 6.5. Most of the decrease in ASNase II activity in the case of CS could be attributed to the low pH of the CS solution (pH = 5.7). TPP was dissolved in DDW, and pH of the resulted solution was about 8.5 which is close to the optimum pH of free ASNase II activity. Thus, the decrease in ASNase II activity may be attributed to the effect of TPP on ASNase II, such as repulsion between the negative charges on TPP and ASNase II, the latter being negatively charged at pH 8.5.

#### Two ways for ASNase II-CSNP preparation

We compared the two methods of preparation of ASNase II-loaded CSNPs through ionotropic gelation method. The entrapment efficiency, size, and zeta potential of the nanoparticles prepared through adding ASNase II-TPP into CS solution were 61%, 143 ± 5 nm, and +35.4 ± 2 mV, whereas they were 68%, 140 ± 4 nm, and +34.9 ± 2 mV when TPP was added into ASNase II-CS solution. No significant differences were seen in the size and zeta potential between the two groups of nanoparticles, but the entrapment efficiency of the nanoparticles which resulted from adding TPP into ASNase II-CS solution was significantly higher than when ASNase II-TPP was added into the CS solution. This observation can be explained by possible interactions of ASNase II molecules with CS polymer before the addition of the cross-linker.

Since proteins are large macromolecules with flexible structure and are able to fold and unfold at different conditions, their interactions with long cationic CS chain and the resulting encapsulation can be complicated, depending on 3-D conformation, electrostatics, and the condition of solution. The polycationic CS chain has a flexible helical conformation in the relatively acidic solution (pH ~ 5.7), due to electrostatic repulsion forces which exist among the protonated amine groups, either within or between polymer chains. The CS chains possess three functional groups for chemical interaction: two hydroxyl groups (primary or secondary) and one primary amine. The negatively charged carboxyl groups on the surface of ASNase II could form electrostatic interactions with the positively charged amine groups and make hydrogen bonds with the hydroxyl groups of the CS chains. Such attachments of a spherical protein molecule did not completely suppress the positive surface charge of CS molecules. Therefore, a high proportion of amine groups on the CS chain might remain free and ready to form cross-links with TPP [29].

As CS is a highly charged polymer at pH ~ 5.7 (below its p*K*_α_ *~* 6.5), it tends to form ion pairs with TPP as a polyvalent anion. At acidic pH, ionotropic cross-linking is the only way of neutralization of protonated CS by TPP ions. Dissolved sodium tripolyphosphate in water dissociates to give both hydroxyl and TPP ions (pH ~ 8.5). OH^−^ and TPP ions in the acidic CS solution could compete to interact with the -NH_3_^+^ of CS, but OH^−^ ions would be immediately neutralized by H^+^ ions and increase the pH of the CS solution. Therefore, by adding TPP, a competition would occur between ionotropic cross-linking by a polyanion and neutralization through deprotonation of CS. Ionotropic cross-linking is an important property which is broadly used in ionotropic gelation processes.

The mild effect of CS on the activity of ASNase II and the higher entrapment efficiency indicated adding TPP into the protein-CS solution as the selected way for nanoparticle preparation in the next steps.

### Optimization of CS and TPP concentrations

CSNPs were prepared by certain amounts of CS (containing 1 mg ASNase II) and TPP. Increasing TPP volume or decrease in CS/TPP ratio led to increased turbidity, indicating a shift in the size variation of the particles to larger dimensions. Optimization of the CS/TPP ratio revealed that when this ratio declined to 0.2/0.06, 0.3/0.08, and 0.4/0.11, high turbidity appeared from the increased aggregation of the nanoparticles. Thus, the CS/TPP ratios of 0.2/0.06, 0.3/0.08, and 0.4/0.11 (Table 1) were discarded because of aggregation which was confirmed microscopically [14,30].

Nanoparticle aggregation occurs under circumstances such as the rise in pH of suspension [31], inadequate speed of homogenization, or high level of cross-linker [29]. López et al. [31] suggested that since the p*K*_α_ value of the chitosan is close to the neutral pH, particles spontaneously aggregate in slightly basic pH, where they become completely uncharged. The final pH of the prepared ASNase II-loaded CSNP suspensions was between 6.2 and 6.3 in all CS/TPP ratios, which was lower than the p*K*_α_ of chitosan. Moreover, increase in TPP concentration might be a more important agent for particle aggregation via cross-linking, as was observed through a raise in TPP volume. Aggregation might be prevented by using a high-speed homogenizer or by sonication during CSNP preparation, but such approaches would lead to inactivation of ASNase and thus could not be used.

Table 3 shows that the average size of the particles increased with a lower CS/TPP ratio (PDI < 0.4) and was positively associated with ASNase II entrapment efficiency. Entrapment efficiency was the highest (70%) when the concentration of CS/TPP was 0.4/0.095. These results might be due to an increased number of interacting units at higher polymer concentrations and to cross-linker levels that lead to the observed increase in particle size and entrapment efficiency [32,33].

**Table 3:** The size, polydispersity index (PDI < 5 and unimodal size distribution), and entrapment efficiency of nanoparticles

**CS (%** ***w*/ *v *)/TPP (%** ***w*/ *v *)**	**Size (nm)**	**PDI**	**EE (%)**
0.2/0.04	138 ± 7	0.35	59.1
0.3/0.06	180 ± 8	0.35	60.2
0.4/0.08	224 ± 10	0.44	62.7
0.2/0.05	187 ± 9	0.43	64.0
0.3/0.075	209 ± 11	0.47	67.3
0.4/0.095	247 ± 10	0.4	70.8

### Optimization of protein loading

Although a general definition identifies nanoparticles as having dimensions below 100 nm, in the area of drug delivery, large nanoparticles (size > 100 nm) may be needed for loading a sufficient amount of a drug onto the particles [34]. According to a working group of the European Science Foundation in 2004, nanoscale in nanomedicine was taken to include active components or objects in the size range from 1 nm to 100 s of nanometers [35]. Accordingly, the CS/TPP ratio of 0.4/0.095 with the highest average entrapment efficiency of 70% and an average size of 247 nm (from the previous step) was applied to optimize protein loading with five different amounts of the lyophilized ASNase II (1, 2, 3, 4, and 5 mg). By adding 5 mg of the lyophilized protein in 1 ml of CS 0.4% (*w*/*v*), a small amount of insoluble precipitate was formed. Therefore, the 5 mg/ml protein concentration was excluded from further study. The average size, zeta potential, protein content, entrapment efficiency, and loading capacity of the ASNase II-loaded CSNPs are displayed in Table 4. At the constant CS/TPP ratio, it seems that there is no sudden change in the particle size. The protein concentration increased from 1 to 4 mg/ml, but about 8% size enlargement of nanoparticles was observed in each step. The final size of nanoparticles with 4 mg/ml of ASNase II was about 36% larger than the corresponding size of nanoparticles in 1 mg/ml.

**Table 4:** **The characteristics of ASNase II-loaded CSNPs prepared by CS/TPP 0.4%/0.095% ( *w */ *v *) and loaded with different amounts of lyophilized ASNase II**

**Lyophilized protein (mg)**	**Size (nm)**	**PDI**	**Zeta potential (mV)**	**Protein content (mg)**	**EE (%)**	**Yeild (mg)**	**LC (%)**
1	250 ± 11	0.48	+35.5 ± 2	0.701 ± 0.011	70.1	3.02	23.3
2	262 ± 10	0.38	+30.7 ± 2	1.464 ± 0.05	73.2	4.18	35.1
3	295 ± 9	0.27	+24.1 ± 3	2.244 ± 0.105	74.8	5.5	40.8
4	340 ± 12	0.42	+21.2 ± 3	3.048 ± 0.07	76.2	6.4	47.6
5	ND	ND	ND	ND	ND	ND	ND

PDI < 5 and unimodal size distribution. ND, not determined (the physicochemical characteristic of the nanoparticles prepared from 5 mg of protein was not suitable for further study); data shown are the mean ± standard deviation.

Entrapment efficiency, yield, and loading capacity of the nanoparticles were increased through an increase in the amount of applied protein. These results are in agreement with those of Yoshida et al. [36] who studied the adsorption of BSA onto ionically cross-linked CS. According to these results, the negatively charged peptide and protein molecules are supposed to be encapsulated more efficiently in a cationic CS polymer. At the pH 5.7, the negatively charged ASNase II molecules (pI ~ 4.9) with their spherical structure could compete with TPP ions to electrostatically react with CS. In other words, ASNase II not only did not interfere with the formation of CSNPs but also might have helped to form CSNPs. The zeta potentials of ASNase II-loaded CSNPs were decreased from +35.5 *±* 2 to +21.2 *±* 3 mV when the protein contents of CSNPs were increased. Decline in the zeta potential could be explained by the reduction in *-*NH_3_^+^ groups on the CS because of further protein loading. In addition to TPP, the negative groups on the surface of ASNase II were counteracted with the positively charged *-*NH_3_^+^ groups of CS during the cross-linking process. Moreover, TPP could counteract with the positively charged *-*NH_3_^+^ groups on the surface of ASNase II and compact the enzyme both inside and on the surface of the particle. Particles possessing a zeta potential of about 20 to 25 mV may sometimes be considered relatively stable [37]. However, having a sufficient zeta potential is extremely important for the role of nanoparticles as carriers for drugs or proteins; the nanoparticles must be capable of ionically holding active molecules or biomolecules. Nanoparticle used for the final characterization were loaded with 4 mg lyophilized ASNase II.

### Fourier transform infrared spectrometry analysis

**Figure 2:**
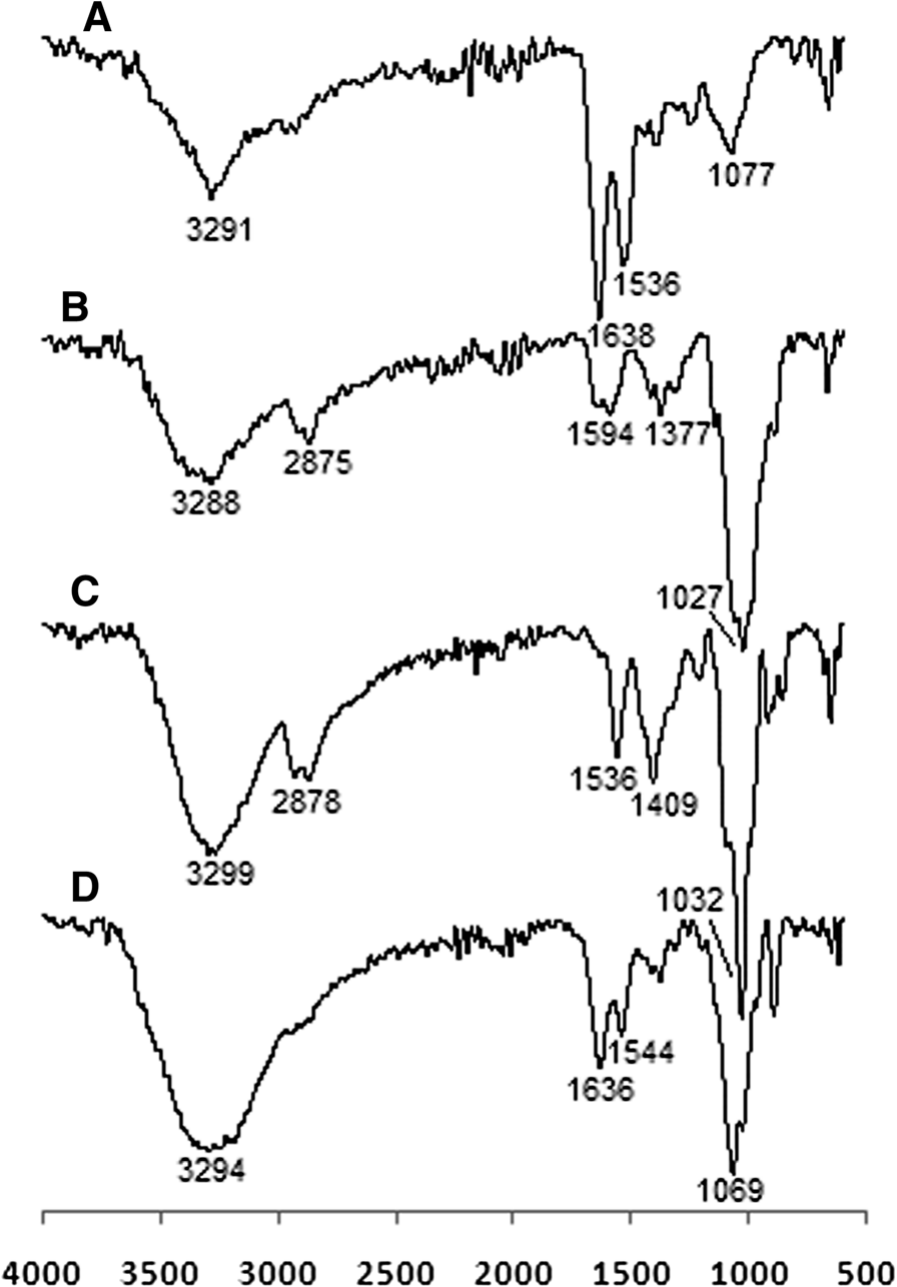
FTIR spectra of (A) ASNase II, (B) CS, (C) CSNPs, and (d) ASNase II-loaded CSNPs.

The FTIR spectra for ASNase II (a), CS (b), CSNPs (c), and ASNase II-loaded CSNPs (d) are shown in Figure 2. The peaks at 3,291 cm^−1^ in the ASNase II spectrum (a) and at 3,288 cm^−1^ in the CS spectrum (b) relate to the stretching of O-H and N-H bonds. In the CSNPs spectrum (c), a shift from 3,288 to 3,299 cm^−1^ is seen and the peak at 3,299 cm^−1^ becomes more intense; this indicates the -NH_3_^+^ interactions with TPP. A corresponding peak in the ASNase II-loaded CSNPs (d) at 3,294 cm^−1^ becomes wider; this effect is attributable to the participation of ASNase II in hydrogen bonding and -NH group interactions [38]. In CSNPs, a new sharp peak appears at 1,409 cm^−1^ and the 1,594 cm^−1^ peak of -NH_2_ bending vibration shifts to 1,536 cm^−1^. We suppose that the phosphoric groups of TPP are linked with -NH_3_^+^ group of CS; inter- and intra-molecular interactions are enhanced in CSNPs [39]. A shift from 1,027 cm^−1^ to the sharper peak at 1,032 cm^−1^ corresponds to the stretching vibration of the P = O groups in CSNPs. Two peaks at 1,636 cm^−1^ (amide I bending) and 1,544 cm^−1^ (amide II bending) in ASNase II-loaded CSNPs correspond to the high intensity peaks at 1,638 and 1,536 cm^−1^ in the ASNase II spectra; this result proves successful loading of ASNase II in CSNPs and also indicates some interactions between CS with TPP and ASNase II [40].

### Morphology studies for the nanoparticles

**Figure 3:**
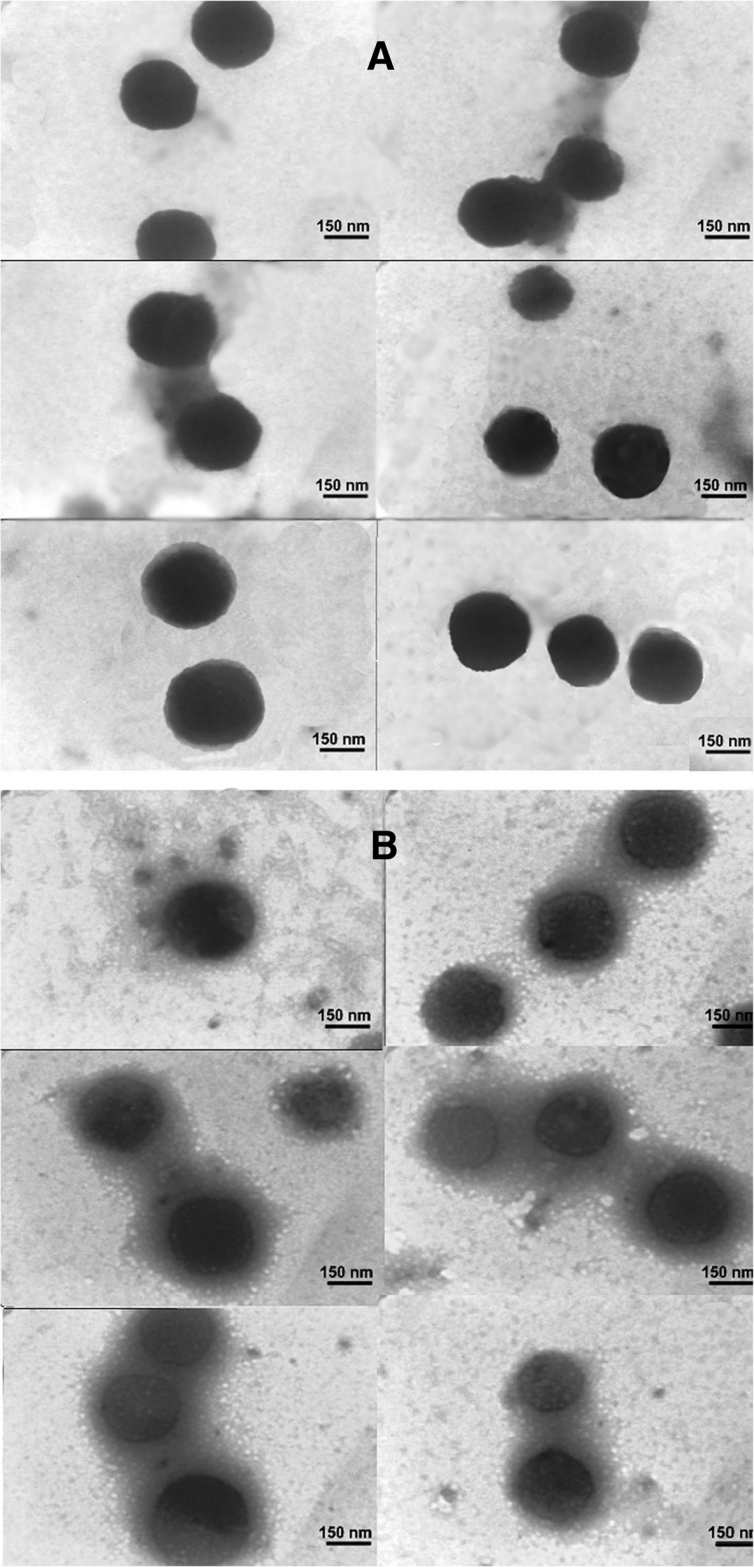
TEM images of CSNPs (A) and ASNase II-loaded CSNPs (B).

Figure 3 shows the TEM images of CSNPs and ASNase II-loaded CSNPs. From the TEM images, both CSNPs (Figure 3A) and ASNase II-loaded CSNPs (Figure 3B) are spherical and exist as discrete spheres, along with a few partial cohesive spheres. The dark core of nanoparticles is due to the fact that the staining reagent has penetrated through the particle. In Figure 3A, a fairly uniform size (the average size 250 ± 11 nm, PDI ~ 0.48) distribution and the smooth border around the CSNPs could be observed. In Figure 3B, ASNase II-loaded CSNPs exhibit an irregular surface with a core surrounded by a fluffy coat made of ASNase II. It can also be noted that the size of the core of the ASNase II-loaded CSNPs (the average size 265 ± 10.5 nm, PDI ~ 0.42) is approximately 6% larger than the particle size of CSNPs. As a consequence, it could be assumed that the significantly increased size of the ASNase II-loaded CSNPs (approximately 333 ± 12.5 nm, PDI ~ 0.47) estimated through TEM and also through DLS (approximately 340 ± 12 nm, PDI ~ 0.42) is due to ASNase II that coated the surface; this would explain the burst release of ASNase II from a huge specific surface area provided by a large number of particles at nanoscale into the buffer during 24 h. The sizes were measured by Manual Microstructure Distance Measurement software.

### *In vitro* ASNase II release

CS forms colloidal particles and entraps bioactive molecules both inside and on the surface of such particles. The mechanisms that have been reported to be involved include chemical cross-linking, ionic cross-linking, and ionic complexation [35]. CS degrades with time in the presence of enzymes (i.e., lysozyme) when inserted into biological environments [41]. However, it has also been found that CSNPs synthesized by ionotropic gelation lose their integrity in aqueous media even in the absence of enzymes. Most drug release profiles from CSNPs exhibit an initial burst release, presumably from the particle surface, followed by a sustained release driven by diffusion of drug through the polymer wall and polymer erosion [10,42]. Gan and Wang [29] investigated the *in vitro* release of BSA from CSNPs. They concluded that the burst is more likely a consequence of rapid surface desorption of large amounts of protein molecules from a huge specific surface area provided by large numbers of particles at nanoscale, and a larger proportion of protein molecules may not be truly embedded in the nanoparticles' inner structure.

**Figure 4:**
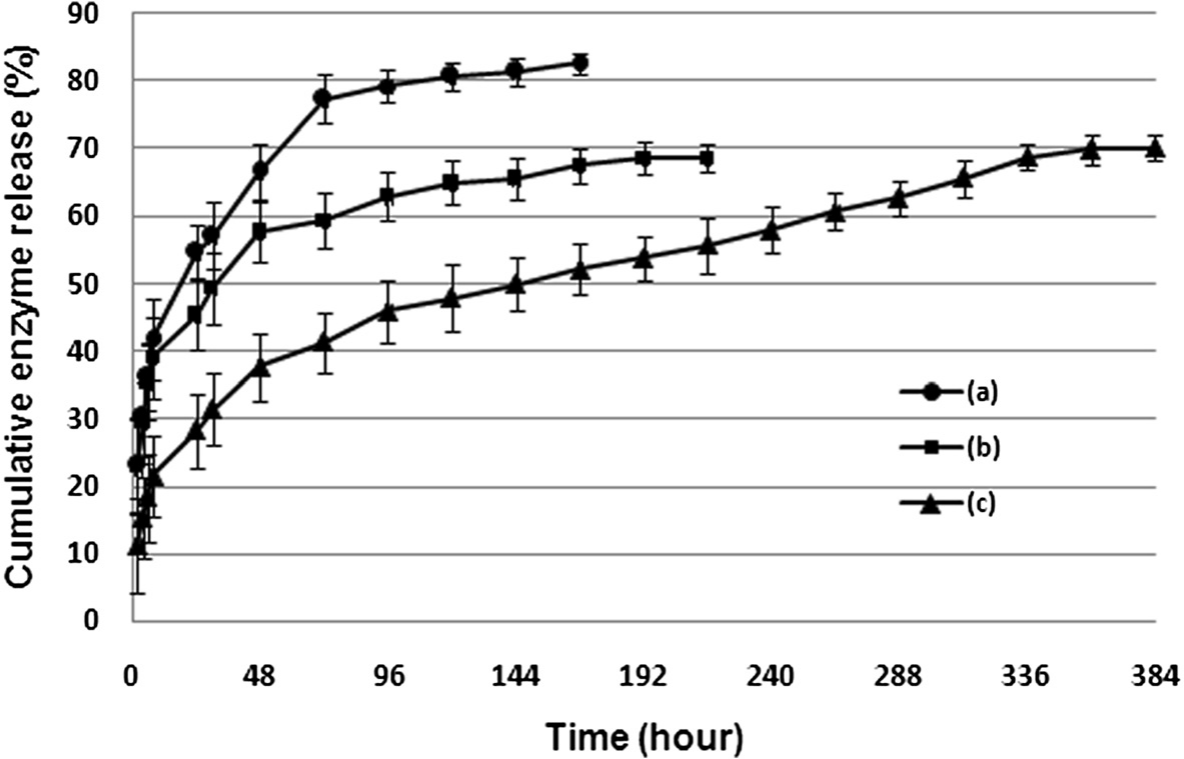
**ASNase II release profiles from the ASNase II-loaded CSNPs in three solutions.** (a) Glycerol (5%)**-**PBS solution (pH 7.4), (b) PBS solution (pH 7.4), and (c) DDW containing 5% glycerol (pH 7.0). CS/TPP of 0.4/0.095 loaded with 4 mg protein.

Figure 4 shows ASNase II release profiles from the ASNase II-loaded CSNPs in three solutions. ASNase II-loaded CSNPs incubated in DDW containing 5% glycerol (pH 7.0) (curve (c)) showed a 28.2% release during 24 h, 39.6% release during 48 h, 54% release during 168 h, and 70% release during 360 h. Curve (a) showed ASNase II release in a 54.7% burst ASNase II release during 24 h, 66.6% release during 48 h, and 82% release during 168 h in glycerol (5%)-PBS solution (7.4). In curve (b), ASNase II showed a 45.3% burst release during 24 h, 57.7% release during 48 h, 68% release during 168 h, and 72% release during 192 h in PBS solution (pH 7.4) without glycerol. Three factors influencing the burst release of ASNase II from CSNPs are hydrogen bonding of glycerol [43], pH of the solution, and ionic strength [31] of PBS. The ASNase II (negatively charged in pH 7 to 7.4) incorporated on the particle surface probably forms a polyelectrolyte barrier. Glycerol, which has hydroxyl groups, could form hydrogen bonds with the hydroxyl groups of ASNase II-loaded CSNPs and prevent the nanoparticles from aggregation by stabilizing them. In addition, the hygroscopic nature of glycerol facilitates penetration of medium inside the polymer matrix that could cause swelling of polymer matrix and lead to the diffusion and release of entrapped ASNase II [44]. Swelling is one of the most important properties of any nanogel. The extent of swelling depends on several external conditions such as pH and ionic strength of the medium [45]. pH is an important parameter in the stability and release of a polypeptide or protein from polymer matrix and depends on cross-link properties [46]. It is known that the p*K*_α_ value of CS is 6.5. The conversion of positively charged amino groups (−NH_3_^+^) of CS into the non-ionized state at a higher pH (>7) value resulted in the reduction of CS cross-linking extent with the counterions (TPP) and then in the increase in swelling of the nanoparticles [25,47]. Structural changes can be introduced by ionic strength variations such as the presence of NaCl (PBS buffer) at low and moderate concentrations, emphasizing the swelling and weakness of CS-TPP ionic interactions, and particle disintegration [31]. This means that its structure can undergo volume phase transitions from swollen to collapsed states and more release of bimolecular drug.

### Effect of pH on free and immobilized enzyme activity and stability

**Figure 5:**
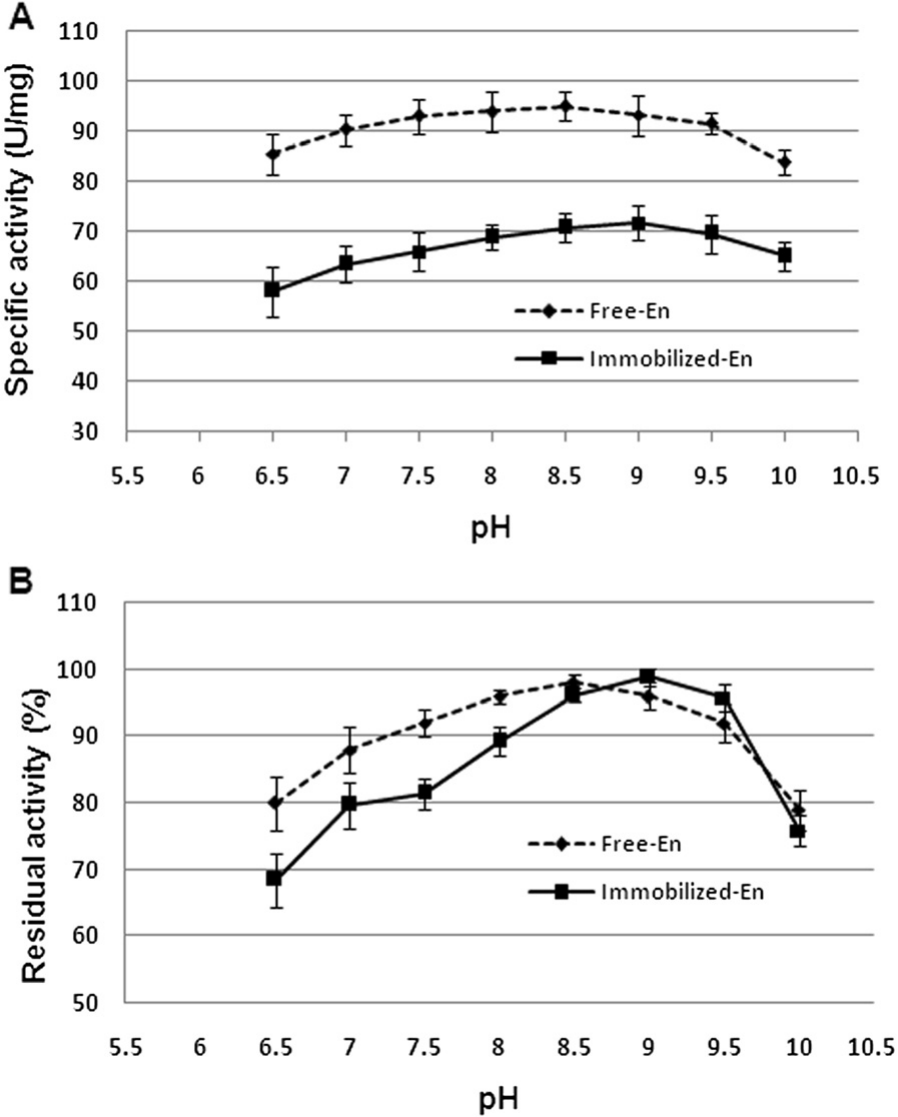
**Effect of pH on the activity (A) and stability (B) of free and immobilized ASNase II.** Activity was measured at standard conditions and compared with untreated control.

ASNase II is an amidohydrolase that is generally active and stable at neutral and alkaline pH. The effect of pH on ASNase II activity and stability of free and immobilized preparations were studied in the range from 6.5 to 10. Figure 5A reveals that the enzymatic activity of both free and immobilized enzyme was optimal in pH 8.5 to 9.0, with a maximum pH 8.5 for the free enzyme and 9 for the immobilized enzyme. The pH stability (Figure 5B) after 24-h incubation at 4°C ± 1°C showed that the free ASNase II retained the maximum of its original activity between pH 8.0 and 9.0 and about 80% at pH 10. The immobilized ASNase II retained about 100% activity at pH 9.0 and about 75% at pH 10.

### The thermostability of the free and immobilized ASNase II

**Figure 6:**
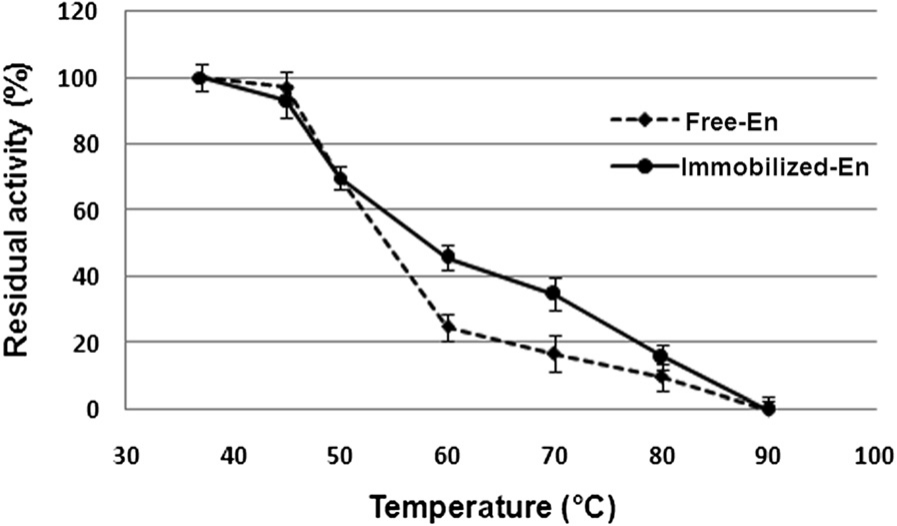
**Effect of temperature on the stability of free and immobilized ASNase II.** After the enzymes were incubated in the buffer solutions (pH 8.5) for 60 min at varying temperatures, the remaining activities were measured at 37°C.

The percentages of the residual activity after 60 min of incubation at 37°C, 45°C, 50°C, 60°C, 70°C, 80°C, and 90°C are shown in Figure 6. The free and immobilized ASNase II were active at temperatures from 37°C to 80°C, with the highest stability at 37°C, but they lost their activities at 90°C. Both forms retained about 70% activity after 60 min of incubation at 50°C, but the process of the loss of activity was faster for the free than immobilized enzyme when the temperature was increased beyond 50°C. As a result, the immobilized ASNase II was more thermostable than the native enzyme.

### *In vitro* half-life of the immobilized and free ASNase II

**Figure 7:**
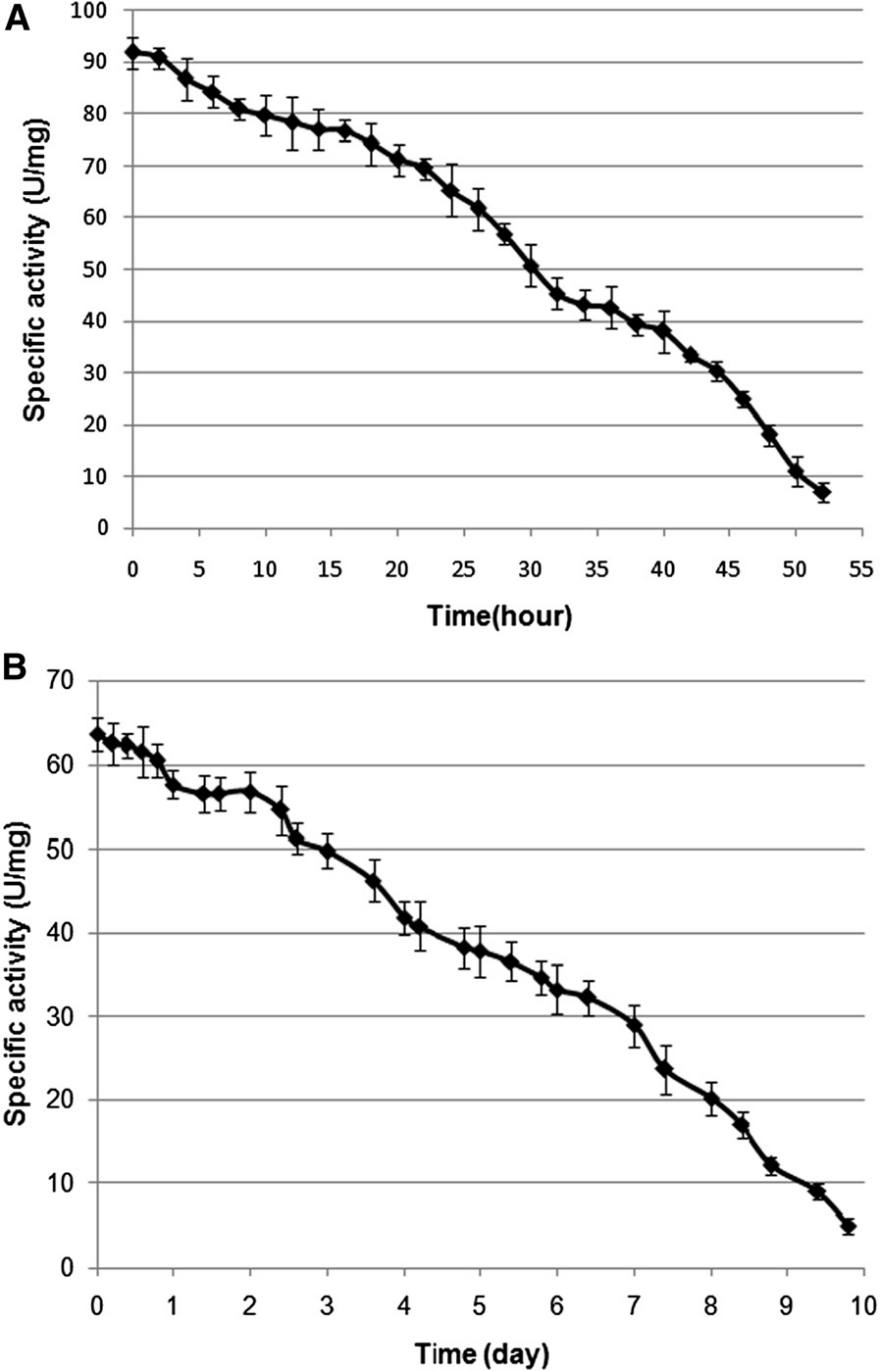
**The *in vitro *** **half-life of the free (A) and immobilized ASNase II (B) in PBS containing 5% glycerol (pH 7.4).**

**Figure 8:**
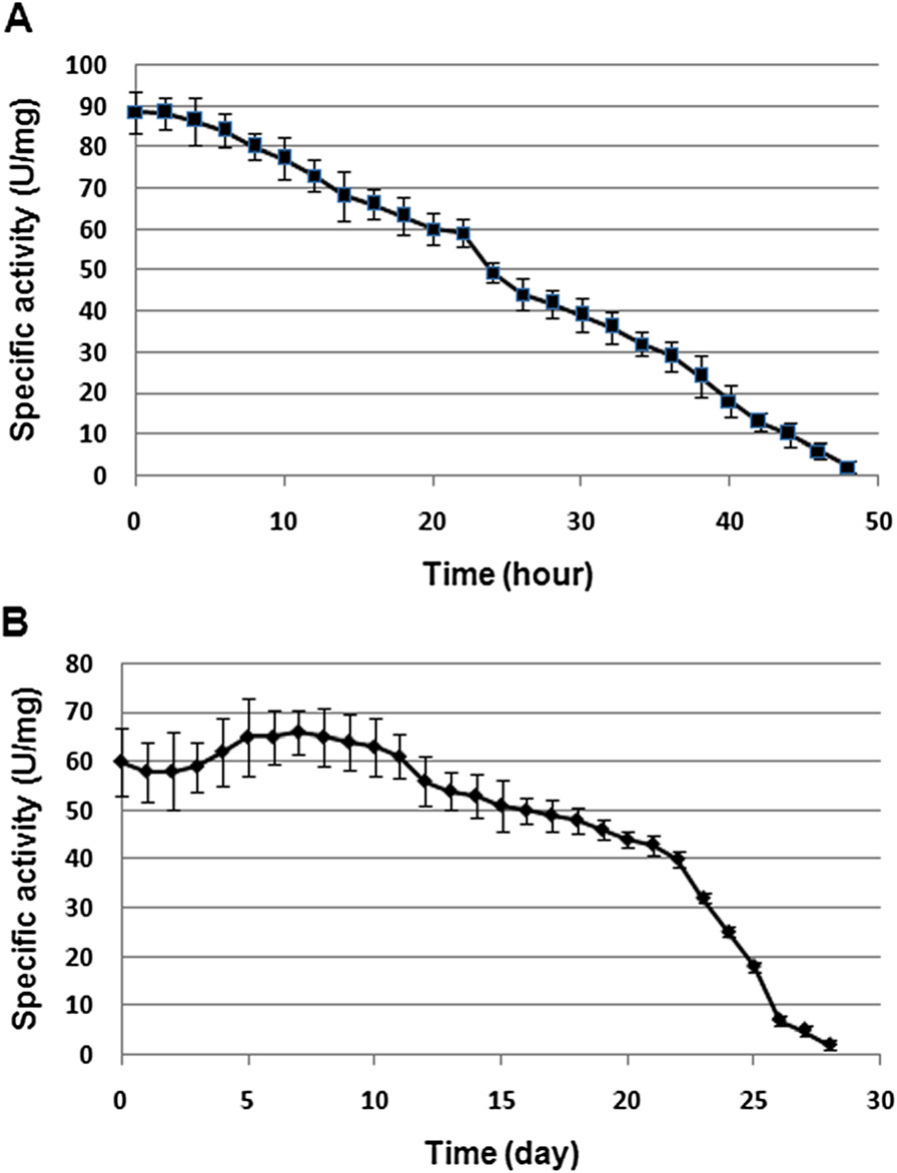
**The *in vitro *** **half-life of the free (A) and immobilized ASNase II (B) in DDW containing 5% glycerol (pH 7.0).**

Solutions of the immobilized and free enzyme in Tris buffer (pH ~ 8.5) containing 5% glycerol were incubated at 37°C to measure the half activity time of both enzymes. Over time, some aggregation of the nanoparticles was observed. DDW containing 5% glycerol (pH ~ 7.0) as the more stable solution and PBS containing 5% glycerol (pH ~ 7.4) as unstable solution for ASNase II-loaded CSNPs were used to measure the half activity time of both enzymes. Both of the immobilized and free enzymes was transferred to the solutions individually and incubated at 37°C. As shown in Figure 7, the half-life of the free enzyme was about 33 h and of the immobilized enzyme about 6.4 days in PBS containing 5% glycerol solution. While in DDW containing 5% glycerol (Figure 8), the half-life of free enzyme (A) decreased to about 26 h, but that of the immobilized enzyme increased to about 23 days. Also, the immobilized enzyme had higher in activity during the 5th to 12th day of incubation in DDW containing 5% glycerol. This effect could be attributed to particle swelling and more penetrating substrate into the particles. The difference in the half-life of ASNase II-loaded CSNPs in the solutions could be attributed to the rate of enzyme release from the nanoparticles. As it was said, ionic strength of PBS helps to the erosion of the nanoparticles and enzyme release. The immobilization of enzymes has been a growing field of research, because it allows an enzyme to catalyze a reaction multiple times with longer half-life and less degradation [42].

The ionotropic gelation method used to prepare ASNase II-loaded CSNPs was so milder than those reported for PLGA [3], hydrogel-magnetic [48] and liposome [7] nanoparticle preparation. Wolf et al. [3] reported that during the ASNase II-PLGA nanosphere preparation, contact with lipophilic interfaces provokes protein denaturation and also necessary shear forces and cavitation stress for the formation of nanodroplets inactivate the enzyme. Gaspar et al. [7] reported that the use of the liposome-encapsulated ASNase II improved the survival of animals with asparagine-dependent P1534 tumors compared with free enzyme. One of the drawbacks of the use of liposomes is the fast elimination from the blood and capture of the liposomal preparations by the cells of the reticulo-endothelial system, primarily in the liver. A number of developments have aimed to reduce this problem, including coating the liposome surface with inert, biocompatible polymers, such as polyethylene glycol (PEG), to slow down the clearance of liposomes [49]. Although PEG remains the gold standard for the steric protection of liposomes [50], it creates an impermeable layer over the liposome surface [51] which could decrease availability of blood asparagines to encapsulated ASNase II. However, research in nanomedicine offers a unique platform for a variety of manipulations that can further enhance the value of the delivered drugs.

## Conclusions

It could be assumed by this study that, when the CSNPs are loaded with hydrophilic macromolecules or drugs, the interactions between them and the gel network can effectively make particles much more stable. The preparation of ASNase II-loaded CSNPs was based on an ionotropic interaction between the positively charged CS and the negatively charged ASNase II and TPP. The negatively charged ASNase II was able to link CS chains electrostatically at pH ~ 5.7 before the addition of the polyanion. Such ASNase II behavior was previously observed in DEAE*-*Sepharose column by positively charged amine groups of DEAE. ASNase II-CS interactions would be strengthened by adding a polyanion and rising pH. So, it could be assumed that CS networks were formed through two cross-linkers of TPP and ASNase II, and the drug itself helped particle formation that is of great interest in pharmaceutical productions. The pH and thermal stability, release, and half-life of ASNase II were evaluated. Compared to the free ASNase II, the immobilized enzyme was more resistant to alkaline pH (8.5 to 9.5) and to high temperatures. ASNase II release could be influenced by pH and the ionic strength of the medium. The immobilized enzyme had an increased half activity time of about 23 days in the low ionic strength solution and about 6.4 days in the high ionic strength solution. This *in vitro* study would provide an impetus for the future *in vivo* investigations. Further studies will be needed to find a suitable particle size and charge, biological responses, and administration route to apply in drug delivery and *in vivo* use.

## Competing interests

The authors declare that they have no competing interests.

## Authors’ contributions

EB drafted the manuscript. KA helped discuss the data analysis. RA, ARM, MTG, and MS organized the final manuscript. All authors read and approved the final manuscript.
